# Characterization of polymorphisms in the follicle-stimulating hormone receptor and insulin-like growth factor-1 genes and their association with fertility traits in Jawa-Brebes cows

**DOI:** 10.14202/vetworld.2023.711-716

**Published:** 2023-04-09

**Authors:** Slamet Hartanto, Agung Budiyanto, Rini Widayanti, Erif Maha Nugraha Setyawan, Imawan Daru Prasetya

**Affiliations:** 1 Department of Reproduction, Obstetrics, and Gynecology, Faculty of Veterinary Medicine, Gadjah Mada University, Yogyakarta, Indonesia; 2 National Research and Innovation Agency (BRIN), Jakarta, Indonesia; 3 Department of Biochemistry and Molecular Biology, Faculty of Veterinary Medicine, Universitas Gadjah Mada, Yogyakarta, Indonesia; 4 Directorate General of Livestock and Animal Health, Ministry of Agriculture, Jakarta, Indonesia

**Keywords:** fecundity, genetic marker, Indonesian cow, restriction fragment length polymorphism-polymerase chain reaction

## Abstract

**Background and Aim:**

The availability of fertility markers is crucial for maintaining, protecting, and improving the genetics of Jawa-Brebes (Jabres) cows. Follicle-stimulating hormone receptor (*FSHR*) and insulin-like growth factor-1 (*IGF-1*) play critical roles in female reproductive physiology. The single-nucleotide polymorphisms (SNPs) *FSHR* *G-278A* and *IGF-1* *C-512T* correlate with cows’ fertility traits. This study aimed to identify these SNPs and their potential associations with fertility parameters in Jabres cows.

**Materials and Methods:**

Samples were collected from 45 heads of multiparous Jabres cows aged 3–10 years with body condition scores of 2.5–5.0 on a 5-point scale in Brebes Regency, Java, Indonesia. These cows were assigned to fertile (n = 16) and infertile groups (n = 29). Polymerase chain reaction (PCR) was carried out for DNA amplification of *FSHR* *G-278A* and *IGF-1* *C-512T* fragments. Restriction fragment length polymorphism-PCR with the restriction enzymes *FaqI* for the product of *FSHR* *G-278A* and *SnaBI* for the product of *IGF-1* *C-512T* was used to identify SNPs.

**Results:**

The *FaqI* enzyme cut the 211 bp DNA fragment of *FSHR* *G-278A* in all samples into two bands of 128 bp and 83 bp (GG genotype). Meanwhile, the genotyping of amplicon products of *IGF-1* *C-512T* generated a single 249 bp fragment (CC genotype) in both groups.

**Conclusion:**

The results showed that the *FSHR G-278A/FaqI* and *IGF-1 C-512T/SnaBI* loci were monomorphic in Jabres cows. Thus, neither *FSHR G-278A/FaqI* nor *IGF-1 C-512T/SnaBI* is a possible genetic marker for fertility in Jabres cows.

## Introduction

Jawa-Brebes (Jabres) cattle, an indigenous Indonesian breed found in Brebes Regency, Java, Indonesia, are renowned for unique reproductive characteristics, including a birth rate of 15–20 calves during the lifespan and good reproductive function when fed inadequate diets [1]. This breed is classified as having a protected and preserved livestock germplasm [2] and plays vital socioeconomic roles [3]. However, there is no effective Jabres breeding program for selecting dams, leading to low pregnancy and calving rates. This contributes to shrinking the population and threatens the survival of Jabres. Indeed, the population of Jabres decreased from 13,890 heads to 12,082 heads in 2021 [4]. There is thus an urgent need for research on genetic markers of fertility to protect, preserve, and enhance the genetics and population of Jabres cows.

Follicle-stimulating hormone (*FSH*) is an essential glycoprotein hormone that critically controls male and female reproductive physiology [5]. It promotes follicular growth and development and facilitates the production of related proteins in parietal granulosa cells in females [6]. Follicle-stimulating hormone exerts its physiological effects through the *FSH* receptor (*FSHR*) [7]. It is mainly found in ovarian granulosa cells in female animals [8–10].

In female livestock, single-nucleotide polymorphisms (SNPs) in the 5’-upstream region (5’-UTR) of the *FSHR* gene have been significantly identified as valuable fertility indicators. For example, significant associations between SNPs in the 5’-UTR of the *FSHR* gene and the litter size of Chinese native ewes have been identified [11, 12]. Moreover, in Chinese Holstein cows, an SNP in the 5’-UTR of the *FSHR* gene (*FSHR G-278A*) was found to correlate with the superovulation response [13]. Furthermore, the *FSHR* *G-278A* mutation was shown to influence service per conception (S/C) in Holstein dairy cows [14].

Meanwhile, insulin-like growth factor-1 (*IGF-1*), which is secreted by liver cells and various other cells, functions as a fundamental growth factor in numerous physiological activities, including reproduction, fetal development, and growth [15–17]. *IGF-1* is crucial in female mammalian fertility because it regulates ovarian function, follicle development, oocyte maturation, and preimplantation embryos [18]. By interacting with gonadotropins, the *IGF-1* gene stimulates the growth and steroidogenesis of ovarian cells, promoting ovarian function [19]. In addition, *IGF-1* inhibits follicular atresia [20].

Based on reported studies, the *IGF-1* gene is a strong putative genetic marker for the reproductive traits of female animals. Insulin-like growth factor-1 polymorphisms have been reported to be correlated with the litter size of Gulin Ma goat and Small Han Tail sheep [21, 22]. In addition, SNPs in the *IGF-1* gene have been shown to influence the fertility rate of Sarda ewes [23]. Mutation of T to C at position 512 in the regulatory region of the *IGF-1* gene (*IGF-1 C-512T*) is the most studied SNP in the *IGF-1* gene associated with fertility traits in cows. This SNP influences the length of the period from calving to commencement of luteal activity (CLA) postpartum, whereby CLA is essential for subsequent pregnancy after calving [24]. The *IGF-1 C-512T* mutation has also been reported to influence calving-first service interval in primiparous Holstein cows [25]. Moreover, several reproductive traits of Holstein cows, such as postpartum estrus and days open, are affected by the SNP *IGF-1* *C-512T* [26].

Restriction fragment length polymorphism-polymerase chain reaction (RFLP-PCR) methods with the *FaqI* restriction enzyme for the *FSHR* gene and the *SnaBI* restriction enzyme for the *IGF-1* gene have been developed to detect fertility markers for dairy cows [13, 14, 24–26].

However, to the best of our knowledge, no study of these has been conducted on Jabres cows. This study aimed to identify *FSHR* *G-278A* and *IGF-1* *C-512T* SNPs and investigate their possible associations with fertility traits, including postpartum estrus, days open, and calving interval (CI), in Jabres cows.

## Materials and Methods

### Ethical approval and Informed consent

All experimental procedures were approved by the Research Ethics Committee of the Faculty of Veterinary Medicine, Universitas Gadjah Mada, Indonesia (00143/EC-FKH/Int./2021). In addition, this research was conducted with the verbal consent of farmers as the cows’ owners.

### Study period and location

The study was carried out from November 2021 to March 2022 on smallholder farmers in the sub-district of Bantar Kawung, Brebes Regency, Central Java Province, Indonesia, and Biochemistry Department, Gadjah Mada University, Yogyakarta, Indonesia.

### Animals

The animals included in this study were multiparous Jabres cows aged 3–10 years with body condition scores of 2.5–5.0 on a 5-point scale. The cows were observed in the sub-district of Bantar Kawung, Brebes Regency, Central Java Province, Indonesia, where the breed originated. The reproductive characteristics recorded and used were postpartum estrus (PPE), days not pregnant (DNP), and CI. The Jabres cows were allocated to two groups: (a) Fertile, defined as PPE of <90 days, DNP of <120 days, and CI of <390 days; and (b) Infertile, defined as EPP of more than 90, DNP of more than 120 days, and CI of more than 390 days. There were 19 Jabres cows in the Fertile group and 26 in the Infertile group. The cows were reared under conditions with management by farmers. Specifically, all cows in this study were raised semi-intensively by smallholder farmers under similar conditions. All cows grazed on native pastures in the morning and were fed rice and corn straw in the afternoon. Water was provided before and after grazing.

### Sample collection and DNA extraction

Blood samples (750 μL) were drawn from the jugular vein of each animal through a 5 mL sterile syringe and put into a 1.5 mL microtube containing 750 μL of absolute ethanol. Blood samples in microtubes were aerated at room temperature (25°C) for 1 d before DNA extraction. Each sample (200 μL) was used for DNA extraction using the gSYNC™ DNA Mini Extraction Kit (Geneaid Biotech Ltd., Taipei, Taiwan), in accordance with the manufacturer’s protocol. Then, DNA samples were kept at −20°C until further molecular analysis.

### Primers

The primers used for *FSHR G-278A* and *IGF-1 C-512T* are shown in Table-1 [14, 24]. Primer synthesis was performed by the Genetika Science Company in Indonesia.

**Table-1:** Primers used for RFLP-PCR of *FSHR* and *IGF-1* gene fragments.

Gene and region	SNP (Genbank accession)	Fragment length	Primers (5’–3’)	Reference
*FSHR* 5’- UTR region	G-278A (GU253337)	211 bp	F – TCCCTGCCCTTCAGTGACGAAC R – AGATACGCCGTCCCCTTTACCT	[14]
*IGF-1* Regulatory region	C-512T (AF017143)	249 bp	F – ATTACAAAGCTGCCTGCCCC R – ACCTTACCCGTATGAAAGGAATATACGT	[24]

RFLP-PCR=Restriction fragment length polymorphism-polymerase chain reaction, FSHR=Follicle-stimulating hormone receptor, *IGF-1*=Insulin-like growth factor-1, SNP=Single-nucleotide polymorphisms

### Polymerase chain reaction reactions

The PCR reaction to amplify *FSHR G-278A* and *IGF-1 C-512T* DNA for all samples comprised a total volume of 50 μL featuring 25 μL of master mix (Bioline My Taq HS Red Mix, 1^st^ Base, Meridian Life Science Inc., London, UK), 20 μL of distilled water, 3 μL of DNA template, and 1 μL (10 pmol) of each primer. The PCR reaction was performed using the Infinigen PCR machine (Biotech Infinigen, CA, USA) with initial denaturation at 94°C for 5 min, followed by 35 cycles at 94°C for 45 s, 55°C for 30 s, and 72°C for 45 s, with final extension at 72°C for 5 min. PCR products were electrophoresed on 1% agarose gel with a 1000 bp DNA ladder (Genaid, Taiwan).

### Genotyping

Restriction fragment length polymorphism-polymerase chain reaction for *FSHR* *G-278A* was carried out in a final volume of 8.15 μL consisting of 2.5 μL of PCR product, 4.5 μL of nuclease-free water, 0.5 μL of 10× Buffer Tango, 0.15 μL of 50× SAM, and 0.5 μL of *FaqI* restriction enzyme (#ER1811, Thermo Fisher Scientific Inc., MA, USA), followed by incubation at 37°C for 16 h. To detect genetic variants of specific base fragments of *IGF-1 C-512T*, reactions were performed using 2.5 μL of PCR product added to 4.5 μL of nuclease-free water, 0.5 μL of 10× Buffer Tango, and 0.5 μL of *SnaBI* restriction enzyme (#ER0401, Thermo Fisher Scientific Inc.), followed by incubation at 37°C for 4 h. The restricted fragments were confirmed by 1.5% agarose gel electrophoresis with a 100 bp DNA ladder (Genaid) and then visualized using an ultraviolet transilluminator (UVP^®^, Fullerton, CA, USA).

Individual genotypes were determined by analyzing the restricted fragment size reported as base pairs. The fragments identified by *FaqI* [GGGAC(10/14)↓] for *FSHR G-278A* were *FaqI* (GG): 128 and 83 bp; *FaqI* (GA): 211, 128, and 83 bp; and *FaqI* (AA) 211 bp (unrestricted). The genotypes identified by *SnaBI* (TAC↓GTA) for *IGF-1 C-512T* were *SnaBI* (TT): 223 and 26 bp; *SnaBI* (CT): 249, 223, and 26 bp; and *SnaBI* (CC): 249 bp (unrestricted). These fragments identified for the *FSHR* and *IGF-1* genes were putative genetic markers for fertility in cows [14, 26].

### Statistical analysis

Restriction fragment length polymorphism-polymerase chain reaction data were analyzed by calculating the allele and genotype frequencies as follows:


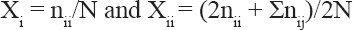


Where X_i_ is the genotype frequency, X_ii_ is the allele frequency, n_ii_ is the number of genotype A_i_A_i_, n_ij_ is the number of genotype A_i_A_j_, and N is the total samples.

## Results

### Restriction fragment length polymorphism-polymerase chain reaction on the *FSHR* gene

**Figure-1:**
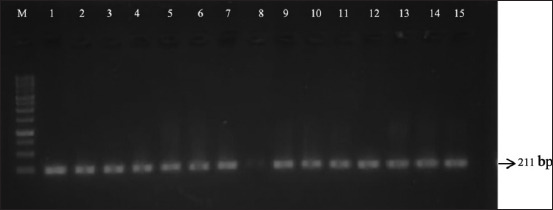
The electrophoresed polymerase chain reaction products of the *FSHR* gene in the Jabres cows. Lane M: Marker. Lanes 1–8: the electrophoresis results of the amplified *FSHR* gene in the infertile group of Jabres cows with a length of 211 bp. Lanes 9–15: the electrophoresis results of the amplified *FSHR* gene in the fertile group of Jabres cows with a length of 211 bp. *FSHR*=Follicle-stimulating hormone receptor.

**Figure-2:**
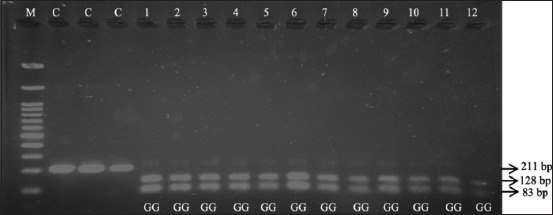
The electrophoresis showing *FSHR* gene products restricted with *FaqI* enzyme in the Jabres cows. Lane M: Marker. Lanes C: PCR products of *FSHR* gene (211 bp) as controls. Lanes 1–6: RFLP-PCR products of *FSHR* gene restricted with *FaqI* (128 bp and 83 bp) in the infertile group of Jabres cows. Lanes 7–12: RFLP-PCR products of *FSHR* gene restricted with *FaqI* (128 bp and 83 bp) in the fertile group of Jabres cows. *FSHR*=Follicle-stimulating hormone receptor, PCR=Polymerase chain reaction, RFLP-PCR=Restriction fragment length polymorphism-polymerase chain reaction.

Figure-1 displays the amplification of 211 bp DNA fragments associated with *FSHR G-278A* of Jabres cows. Restriction fragment length polymorphism-polymerase chain reaction with the *FaqI* restriction enzyme was performed to cut the mutation point of the amplified DNA fragment of *FSHR G-278A*. The enzyme cut this fragment into two bands of 128 and 28 bp in all samples from both groups, as demonstrated in Figure-2. This confirmed that all of the studied samples were monomorphic and genotyped as GG genotypes (Table-2).

**Table-2:** Genotype and allele frequencies of *FSHR* gene in Jabres cows.

Group	N (heads)	Genotype frequency			Allele frequency	
	
		GG	GA	AA	G	A
Fertile	19	01.00	00.00	00.00	01.00	00.00
Infertile	26	01.00	00.00	00.00	01.00	00.00

GG and AA=Homozygous genotypes, GA=Heterozygous genotypes, G and A=Alleles, *FSHR*=Follicle-stimulating hormone receptor

### Restriction fragment length polymorphism-polymerase chain reaction on the *IGF*-1 gene

**Figure-3:**
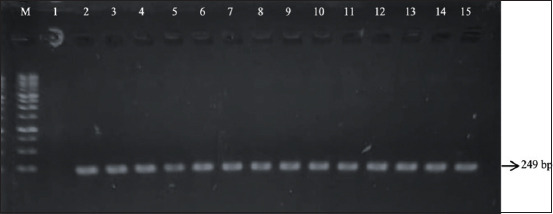
The electrophoresis figure displaying PCR product (249 bp) of *IGF-1* in the Jabres cows. Lane M: Marker. Lanes 1–8: 249 bp PCR product of *IGF-1* gene in the infertile group of Jabres cows. Lanes 9–15: 249 bp PCR product of *IGF-1* gene in the fertile group of Jabres cows. PCR=Polymerase chain reaction, *IGF-1=*Insulin-like growth factor-1.

**Figure-4:**
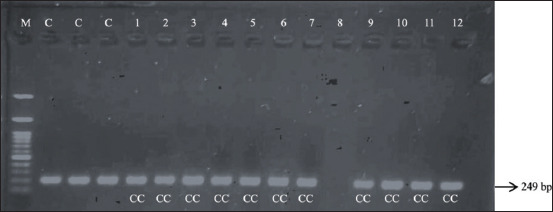
Gel electrophoresis displaying the *IGF-1* gene products restricted with *SnaBI* enzyme in the Jabres cows. Lane M: Marker. Lanes C: PCR products of *IGF-1* gene (249 bp) as controls. Lanes 1–6: 249 bp RFLP-PCR products of *IGF-1* gene restricted with *SnaBI* enzyme in the infertile group of Jabres cows. Lanes 7–12: 249 bp RFLP-PCR products of *IGF-1* gene restricted with *SnaBI* enzyme in the fertile group of Jabres cows. *IGF-1=*Insulin-like growth factor-1, PCR=Polymerase chain reaction, RFLP-PCR=Restriction fragment length polymorphism-polymerase chain reaction.

This study showed that the PCR amplification of *IGF-1 C-512T* produced a 249 bp DNA fragment, as displayed in Figure-3. The *SnaBI* restriction enzyme was used for genotyping amplicon products. However, the specific DNA-cutting sites of *IGF-1 C-512T* were left uncut by the *SnaBI* enzyme in all Jabres cows of both groups. Therefore, the genotyping of amplicon products of *IGF-1 C-512T* in Jabres cows generated a single 249 bp fragment, as shown in Figure-4. This indicated that all of these Jabres cows possessed the same genotype, namely, the CC genotype (Table-3).

**Table-3:** Genotype and allele frequencies of *IGF-1* gene in Jabres cows.

Group	N (heads)	Genotype frequency			Allele frequency	
	
		CC	CT	TT	C	T
Fertile	19	01.00	00.00	00.00	01.00	00.00
Infertile	26	01.00	00.00	00.00	01.00	00.00

CC and TT=Homozygous genotypes, CT=Heterozygous genotypes, C and T=Alleles, *IGF-1*=Insulin-like growth factor-1

## Discussion

Follicle-stimulating hormone receptor is necessary for ovarian follicle development and ovulation [27]. Several signaling pathways that promote follicle development and estrogen synthesis are activated by *FSHR* [28]. Numerous studies have shown that *FSHR* mutations are indicators of fertility and infertility during female reproduction. In addition, the SNP *FSHR* rs6166 is a genetic indicator of premature ovarian insufficiency (POI) in Asian women [29]. Folliculogenesis failure caused by the mutation of the *FSHR* gene led to premature ovarian insufficiency in women [30]. Moreover, Lindgren *et al*. [31] discovered that the *FSHR* variant N680S is an excellent predictor of pregnancy probability in female patients undergoing *in vitro* fertilization.

Single-nucleotide polymorphisms in the *FSHR* gene are also significantly associated with ovarian activity in cows. For example, Yang *et al*. [13] reported that an SNP in the 5’-UTR region of *FSHR* (*FSHR* G-278A) is correlated with superovulation response, including the total number of ova and the number of transferable embryos, in Chinese Holstein cows. Single-nucleotide polymorphisms in the coding region (c.337C>G, c.871A>G and c.1973C>G) of the *FSHR* gene also influence the numbers of embryos yielded and unfertilized oocytes in Holstein cows [32]. Furthermore, Hirayama *et al*. [33] demonstrated that the *FSHR* SNP c.337C>G affected the number of embryos produced by superovulation in Japanese Black cattle. This variant was also shown to affect the ovulation rates in beef heifers [34].

Although the *FSHR* gene is crucial in reproduction and fertility traits, we discovered that the studied locus of *FSHR G-278A* in the 5’-UTR in the fertile and infertile Jabres groups was monomorphic. No variation in the *FSHR G-278A/SnaBI* locus was found in any Jabres cows. Follicle-stimulating hormone receptor *G-278A* appears to be highly conserved within the investigated Jabres cow groups. This may be due to the high inbreeding coefficient of the investigated population or the presence of a connection between the sampled animals. It has been reported that uncontrolled mating resulted in highly inbred Jabres cattle [35]. Thus, the *FSHR G-278A/SnaBI* locus does not affect fertility traits in Jabres cows.

Our result is similar to the finding of Abeygunawardana *et al*. [36], who reported that the *FSHR G-278A/SnaBI* locus had no association with fertility traits in crossbred multiparous dairy cows (*Bos indicus* × *Bos taurus*). Moreover, because no variation was found, it was reported that the *FSHR* gene was not correlated with reproductive parameters, including ovarian hypofunction in Madrasin cattle [37]. However, these findings contrast with the results of Sharifiyazdi *et al*. [14], who found that the *FSHR G-278A/SnaBI* locus affected the reproductive traits of Holstein dairy cows, such as S/C. An *FSHR* SNP at *A-320T/TaqI* of the 5’-UTR region was also reported to be associated with S/C in Antioquia Holstein cattle [38].

*IGF-1* is a central regulator of many intraovarian activities during follicular development [39]. It promotes follicular development by regulating the proliferation and differentiation of granulosa cells, steroid synthesis, and gonadotropin stimulation [40]. Moreover, granulosa cells regulate oocyte development in ovarian follicles by synthesizing steroids and growth factors [41]. SNPs in *IGF-1* have been reported to be closely associated with fertility in female livestock [21–23, 42].

In our investigation, genotyping of *IGF-1 C-512T* with the *SnaBI* enzyme generated a single CC genotype with a DNA fragment of 249 bp in all Jabres cows. Consequently, only the C allele was discovered and no T allele was detected. Although our study included few samples, this work indicated no association between the SNP *IGF-1 C-512T* and reproductive traits in Jabres cows. Anggraeni *et al*. [43] also discovered no correlation between intron 1 of the *IGF-1/SnaBI* locus and fertility traits in Peranakan Ongole cows. The genotype of *IGF-1 C-512T/SnaBI* was also reported not to affect the fertility of Holstein cows reared semi-intensively or intensively [44]. However, Silveira *et al*. [26] found that the SNP *IGF-1 C-512T/SnaBI* correlated with fertility parameters of Holstein cows, including postpartum estrus and days open. Moreover, the SNP *IGF-1 C-512T/SnaBI* was found to influence CLA as a crucial factor for the following pregnancy after calving [24] and the calving-first service interval in Holstein cows [25].

## Conclusion

Neither *FSHR G-278A/FaqI* nor *IGF-1 C-512T/SnaBI* is a possible genetic marker for fertility in Jabres cows. Due to the crucial roles of the *FSHR* and *IGF-1* genes, further study is needed to search for other loci within these essential genes in Jabres cows. A study with a larger sample size is also necessary to confirm the effect of specific SNPs of the *FSHR* and *IGF-1* genes on the reproductive characteristics of Jabres cows.

## Authors’ Contributions

SH and AB: Designed the study and wrote, edited, and revised the manuscript. SH and RW: Conducted study in the laboratory. SH, AB, and RW: Analyzed and interpreted the data. AB, RW, and EMNS: Supervised the study and reviewed the manuscript. SH and IDP: Collected the data and blood samples. All authors have read, reviewed, and approved the final manuscripts.
